# Joint association of triglyceride-glucose index and atherogenic lipid markers with incident stroke risk

**DOI:** 10.1038/s41598-026-56940-5

**Published:** 2026-06-12

**Authors:** Laiang Yao, Guiling Sun, Lanju Zhang, Xiangming Xu, Yan Zhang

**Affiliations:** 1 Linyi People’s Hospital, Linyi, China; 2 Department of Epidemiology and Biostatistics, Joe C Wen School of Population and Public Health, University of California, Irvine, USA

**Keywords:** Stroke, Tryglycerdie glucose index, Insulin resistance, Non-HDL cholesterol, Remnant cholesterol, Cerebrovascular diseases, Biomarkers, Cardiology, Diseases, Health care, Medical research, Neurology, Risk factors

## Abstract

The triglyceride-glucose (TyG) index and atherogenic cholesterol markers, including non-HDL cholesterol and remnant cholesterol, are significant predictors of atherosclerotic cardiovascular disease (ASCVD). However, the joint effects and predictive value of TyG and atherogenic cholesterol markers for incident stroke remain insufficiently understood. We included participants from the China Health and Retirement Longitudinal Study (CHARLS) enrolled at baseline in 2011 and followed them through 2020. Participants were categorized into four groups according to the median values of the TyG index and each cholesterol marker, with those having both values below the median serving as the reference group. Cox proportional hazards models were used to evaluate the independent and joint associations of TyG and atherogenic cholesterol markers with incident stroke. Restricted cubic spline models were applied to assess dose–response relationships, and receiver operating characteristic (ROC) curve analyses were used to examine predictive performance. A total of 8,544 participants were included (mean [SD] age 59.0 [9.5] years; 52.1% female). 522 incident stroke events occurred during a maximum follow-up of 9.0 years. TyG, non-HDL cholesterol and remnant cholesterol were all independently associated with stroke risk. Compared with participants with both TyG and cholesterol markers below median, those with both markers above median had the greatest stroke risk in fully adjusted models (HR for high TyG and high non-HDL cholesterol: 1.76 [95% CI 1.41–2.20]; HR for high TyG and high remnant cholesterol: 1.45 [95% CI 1.18–1.77]). Adding TyG and each cholesterol marker to traditional risk factors modestly improved risk discrimination, with the model combining TyG and non-HDL cholesterol yielding the highest AUC. The TyG index and atherogenic cholesterol markers were independently and jointly associated with increased stroke risk among middle-aged and older adults. Adding these routinely available metabolic and lipid markers modestly improved discrimination for incident stroke.

**Supplementary Information:**

The online version contains supplementary material available at 10.1038/s41598-026-56940-5.

## Introduction

Stroke is a major global health concern and a leading cause of mortality and long-term disability worldwide^1,2^. The burden is particularly high among middle-aged and older adults, and a substantial proportion of stroke events is attributable to modifiable metabolic and vascular risk factors^2,3^. Therefore, identifying accessible biomarkers that capture early cardiometabolic and atherosclerotic risk may help improve primary prevention in aging populations. The triglyceride–glucose (TyG) index, derived from fasting triglyceride and glucose levels, is widely used as a simple surrogate marker of insulin resistance and has been associated with cardiometabolic disorders and atherosclerotic cardiovascular outcomes^4,5^. In parallel, atherogenic lipid markers, such as non-HDL cholesterol and remnant cholesterol, have gained increasing attention because they reflect cholesterol carried by atherogenic lipoproteins beyond conventional LDL cholesterol and may capture residual vascular risk^6,7^.

Although TyG and atherogenic lipid markers have each been linked to cardiovascular and cerebrovascular risk, their joint contribution to incident stroke remains insufficiently understood. Recent studies have examined related biomarker combinations, such as TyG with non-HDL cholesterol for coronary heart disease or TyG with remnant cholesterol for carotid plaque^8,9^. However, these studies were often conducted in selected clinical populations and focused primarily on coronary disease, carotid plaque, or other surrogate atherosclerotic outcomes rather than incident stroke. Thus, evidence from large general population-based longitudinal cohorts remains limited. It is also unclear whether combining TyG with non-HDL cholesterol or remnant cholesterol provides incremental discriminatory information for incident stroke beyond traditional risk factors.

To address this gap, we used data from the China Health and Retirement Longitudinal Study, a nationwide longitudinal cohort of middle-aged and older Chinese adults, to examine the independent and joint associations of the TyG index and atherogenic lipid markers with incident stroke. We also explored whether adding these biomarkers, individually and jointly, to a traditional risk-factor model would improve discrimination for incident stroke. By focusing on routinely available laboratory measures , this study aimed to clarify whether combined metabolic and atherogenic lipid assessment may help identify individuals at higher risk of stroke in a general aging population.

## Methods

### Study design and study population

The China Health and Retirement Longitudinal Study (CHARLS) is an ongoing, nationally representative cohort of middle-aged and older adults in China designed to investigate the social, economic, and health determinants of aging. At baseline (2011, wave 1), CHARLS recruited 17,708 participants from 28 provinces using a multistage, stratified, systematic sampling strategy, with follow-up surveys conducted in 2013 (wave 2), 2015 (wave 3), 2018 (wave 4), and 2020 (wave 5). Trained interviewers administered standardized questionnaires to collect information on sociodemographic characteristics, lifestyle factors, and health status. The detailed study design, objectives, and cohort profile have been described elsewhere^10^. The CHARLS protocol was approved by the Biomedical Ethics Review Committee of Peking University, and written informed consent was obtained from all participants in each survey wave. Our study followed the Strengthening the Reporting of Observational Studies in Epidemiology (STROBE) guidelines.

**Fig. 1:**
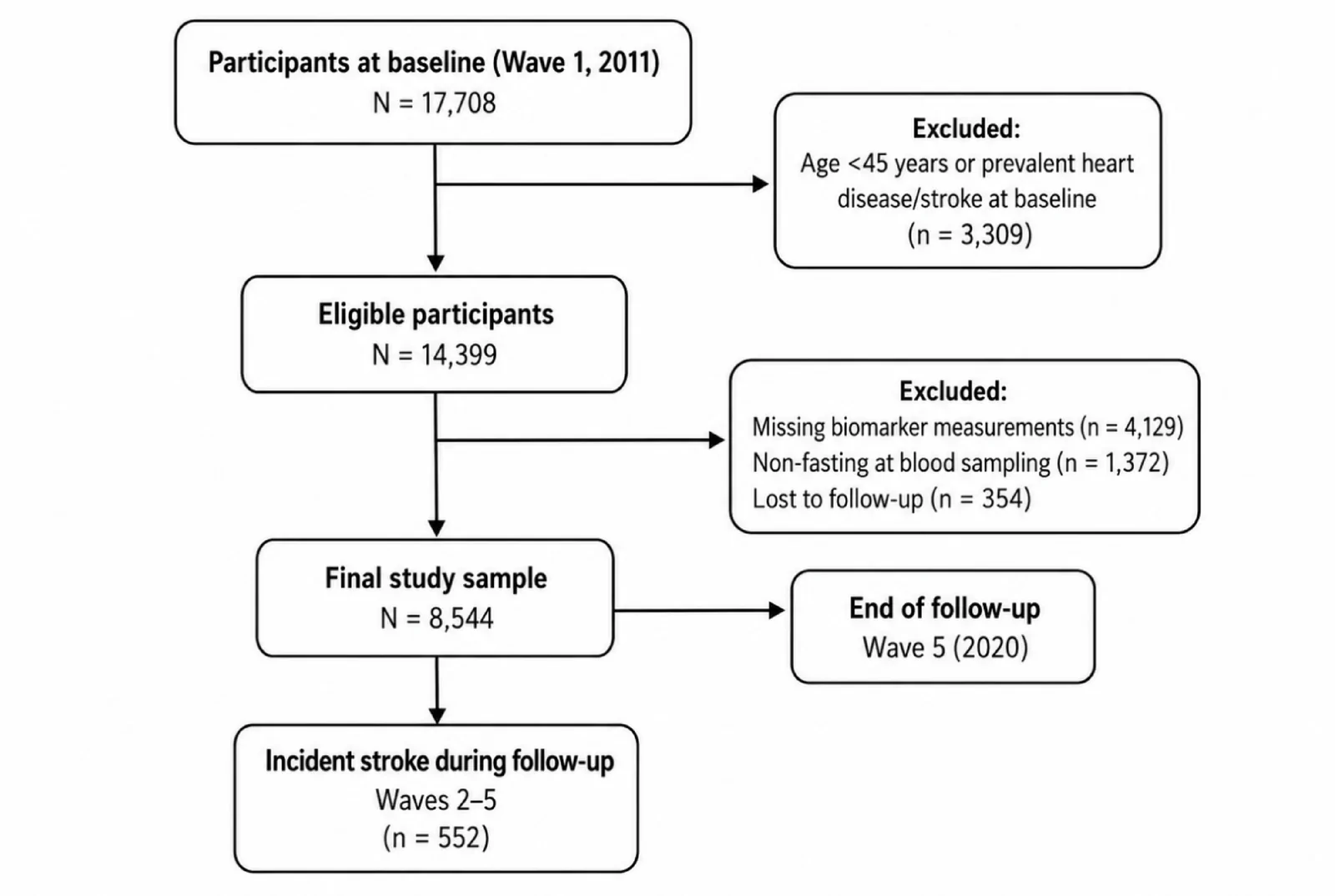
Selection process of participants from CHARLS 2011–2020.

The participant selection process is summarized in Fig. 1. For the present analysis, we included participants enrolled in the baseline survey (wave 1) and followed them across the subsequent four waves. Of the 17,708 baseline participants, we excluded those younger than 45 years and those with prevalent stroke or heart disease at baseline. In CHARLS, prevalent heart disease was based on self-reported physician diagnosis of heart attack, coronary heart disease, congestive heart failure, or other heart problems. We further excluded participants with missing blood measurements, participants who were non-fasting at the time of blood sampling and those lost at follow up, resulting in a final analytic sample of 8,544 participants.

### Exposures

The TyG index, non-HDL cholesterol, and remnant cholesterol were assessed using fasting blood samples collected at Wave 1, the baseline survey in 2011. Fasting venous blood samples were collected by trained medical staff from the Chinese Center for Disease Control and Prevention using a standardized protocol and were analyzed at a central laboratory. Blood lipids and glucose were measured using enzymatic colorimetric methods. Atherogenic cholesterol markers, including non–high-density lipoprotein cholesterol (non–HDL-C) and remnant cholesterol, were derived using standard formulas from previous studies^11,12^. Non–HDL-C was calculated as total cholesterol minus high-density lipoprotein cholesterol (HDL-C). Remnant cholesterol was calculated as total cholesterol minus HDL-C minus low-density lipoprotein cholesterol (LDL-C). The triglyceride–glucose (TyG) index was calculated as Ln [triglycerides (mg/dL) × glucose (mg/dL) / 2], consistent with prior literature^13,14^.

### Covariates

Covariates included sociodemographic characteristics (age, sex, educational level, marital status, and urban/rural residency), health behaviors (smoking and alcohol consumption), and clinical factors (diabetes, hypertension, dyslipidemia, body mass index [BMI], systolic blood pressure [SBP], C-reactive protein, and hemoglobin A1c [HbA1c]) were assessed at baseline. Educational level was categorized as “Below high school,” “High school,” or “College or above.” Marital status was classified as “Married” or “Other.” Smoking status was categorized as current smoker or former/never smoker, and drinking status as current drinker or former/never drinker. BMI was calculated as weight in kilograms divided by height in meters squared (kg/m^2^), and BMI ≥ 24.0 kg/m^2^ was classified as overweight according to Asian-specific criteria^15^. Hypertension was defined as SBP ≥ 140 mmHg and/or diastolic blood pressure ≥ 90 mmHg, self-reported physician diagnosis of hypertension, or current use of antihypertensive medication. Diabetes was defined as fasting plasma glucose ≥ 7.0 mmol/L, self-reported physician diagnosis of diabetes, or current use of antidiabetic medication. Dyslipidemia was defined as abnormal levels of total cholesterol, LDL-C, HDL-C, or triglycerides, self-reported physician diagnosis of dyslipidemia, or current use of lipid-lowering medication^16^.

### Assessment of outcome

The primary outcome was first incident stroke during follow-up (waves 2–5), ascertained through self-reported physician diagnosis at follow-up interviews. Participants were classified as having experienced a stroke if they answered “yes” to the question, “Have you been diagnosed with a stroke by a doctor?” The first survey wave at which a doctor-diagnosed stroke was reported was taken as the time of the incident event. For participants who reported stroke during follow-up, only the first reported stroke event was included, and recurrent stroke events were not considered. When the exact date of stroke onset was unavailable, the interview date of the wave at which stroke was first reported was used as the event date.

Follow-up began after baseline assessment in 2011 and continued until the first occurrence of incident stroke, death, or censoring, whichever came first. The censoring date was defined as the date of the last completed survey for each participant, with follow-up available through wave 5 (2020). Information on death was ascertained up to the last survey wave in CHARLS.

### Statistical analysis

Baseline characteristics were summarized using means and standard deviations (SD) or medians and interquartile ranges (IQR) for continuous variables, and counts with percentages for categorical variables. Differences across exposure categories were assessed using analysis of variance (ANOVA) or Kruskal–Wallis rank-sum tests for continuous variables, as appropriate, and χ^2^ tests or Fisher’s exact tests for categorical variables. Missing covariate data were handled using multiple imputation by chained equations (MICE)^17^. The proportions of missing data for each covariate are presented in Supplementary Tables 2–3.

Cox proportional hazards regression models were used to estimate hazard ratios (HRs) and 95% confidence intervals (CIs) for the associations of the TyG index, atherogenic lipid markers, and their joint categories with incident stroke. For joint effect analyses, the TyG index, non–HDL cholesterol, and remnant cholesterol were each dichotomized at their median values. For each pairwise combination (TyG with non–HDL cholesterol and TyG with remnant cholesterol), participants were categorized into four groups: (1) both TyG and the cholesterol marker below the median (reference group); (2) TyG above the median and the cholesterol marker below the median; (3) TyG below the median and the cholesterol marker above the median; and (4) both TyG and the cholesterol marker above the median. Two models were fitted for each exposure. Model 1 was unadjusted. Model 2 was adjusted for prespecified covariates selected a priori based on clinical relevance and previous literature, including age, sex, educational level, marital status, residency, smoking status, drinking status, BMI, SBP, C-reactive protein, and HbA1c. The proportional hazards assumption was assessed using Schoenfeld residuals. Potential nonlinear dose–response relationships between the TyG index, atherogenic cholesterol markers, and incident stroke were evaluated using restricted cubic splines (RCS).

To explore the incremental discriminatory value of the TyG index and atherogenic lipid markers, we conducted ROC curve analyses using a traditional risk-factor model as the reference model. The reference model included age, sex, educational level, marital status, residency, smoking status, drinking status, BMI, SBP, C-reactive protein, and HbA1c. We then added the TyG index, non-HDL cholesterol, and remnant cholesterol individually and jointly to assess changes in AUC. These analyses were intended to evaluate incremental discrimination beyond available conventional risk factors.

Several sensitivity analyses were conducted to assess the robustness of our findings. (1) We repeated all main analyses using complete-case data without multiple imputation. (2) To reduce potential reverse causation, we repeated the analyses after excluding participants who developed stroke within the first two years of follow-up. (3) We further adjusted the models for use of antihypertensive, glucose-lowering, and lipid-lowering medications. (4) To account for the competing risk of death, we additionally performed Fine–Gray competing risk models, treating incident stroke as the event of interest and death before stroke as the competing event. (5) To more finely evaluate and verify the joint associations of the TyG index and atherogenic cholesterol markers, we reclassified the TyG index, non–HDL cholesterol, and remnant cholesterol into tertiles and constructed joint exposure categories. For each pairwise combination, participants were grouped into six joint categories derived from the cross-classification of tertiles of TyG or the corresponding cholesterol marker, with the group having both biomarkers in the lowest tertile specified as the reference.

In addition, we performed stratified analyses by sex (male vs. female), age group (< 65 vs. ≥ 65 years), and BMI status (< 24 vs. ≥24 kg/m^2^). All data preprocessing, statistical analyses, and visualizations were conducted using RStudio (version 4.4.3). All p-values were two-sided, and a p-value < 0.05 was considered statistically significant.

## Results

### Baseline characteristics of the participants

A total of 8,544 participants were included in the final analysis. The mean (SD) age at baseline was 59.0 (9.5) years, and 52.1% were female. The median value of TyG, non-HDL cholesterol and remnant cholesterol were 8.57, 138.40 mg/dL and 18.94 mg/dL, respectively. Over a maximum follow-up of 9.0 years, 552 incident stroke events were documented. Baseline characteristics according to the joint categories of the TyG index and atherogenic cholesterol markers are presented in Table 1 and Supplementary Table 1. Compared with participants who had both the TyG index and non–HDL cholesterol below the median (*n* = 2,884), those with both markers at or above the median (*n* = 2,863) were more likely to be women, to reside in urban areas, and to be non-smokers and nondrinkers. Similar patterns were observed when participants were classified jointly by the TyG index and remnant cholesterol. Participants with both biomarkers at or above the median were also more likely to have hypertension, diabetes, or dyslipidemia (*p* < 0.001 for all). Baseline characteristics for participants without multiple imputation are also presented in Supplementary Tables 4 and 5.

**Table 1:** Baseline characteristics of participants based on TyG and non-HDL cholesterol status.

	Level	Overall	Reference group	Non HDL-C above median	TyG above median	Both above median	*p*-value
*N*		8544	2884	1394	1403	2863	
Age (mean (SD))		59.03 (9.45)	59.08 (9.80)	59.23 (9.37)	58.73 (9.36)	59.04 (9.18)	0.551
Sex (%)	Female	4448 (52.1)	1310 (45.4)	742 (53.2)	723 (51.5)	1673 (58.4)	< 0.001
	Male	4096 (47.9)	1574 (54.6)	652 (46.8)	680 (48.5)	1190 (41.6)	
Education (%)	Below High School	7648 (89.5)	2607 (90.4)	1251 (89.7)	1240 (88.4)	2550 (89.1)	0.274
	College or above	108 (1.3)	34 (1.2)	12 (0.9)	23 (1.6)	39 (1.4)	
	High school	788 (9.2)	243 (8.4)	131 (9.4)	140 (10.0)	274 (9.6)	
Marry (%)	Marry	7546 (88.3)	2551 (88.5)	1236 (88.7)	1229 (87.6)	2530 (88.4)	0.819
	Other	998 (11.7)	333 (11.5)	158 (11.3)	174 (12.4)	333 (11.6)	
Residency (%)	Rural	5466 (64.0)	1995 (69.2)	915 (65.6)	866 (61.7)	1690 (59.0)	< 0.001
	Urban	3078 (36.0)	889 (30.8)	479 (34.4)	537 (38.3)	1173 (41.0)	
Current smoker (%)	No	5958 (69.7)	1879 (65.2)	988 (70.9)	976 (69.6)	2115 (73.9)	< 0.001
	Yes	2586 (30.3)	1005 (34.8)	406 (29.1)	427 (30.4)	748 (26.1)	
Current drinker (%)	No	5576 (65.3)	1781 (61.8)	917 (65.8)	918 (65.4)	1960 (68.5)	< 0.001
	Yes	2968 (34.7)	1103 (38.2)	477 (34.2)	485 (34.6)	903 (31.5)	
Hypertension (%)	No	6553 (76.7)	2391 (82.9)	1135 (81.4)	1034 (73.7)	1993 (69.6)	< 0.001
	Yes	1991 (23.3)	493 (17.1)	259 (18.6)	369 (26.3)	870 (30.4)	
Dyslipidemia (%)	No	7843 (91.8)	2752 (95.4)	1312 (94.1)	1286 (91.7)	2493 (87.1)	< 0.001
	Yes	701 (8.2)	132 (4.6)	82 (5.9)	117 (8.3)	370 (12.9)	
Diabetes (%)	No	8095 (94.7)	2823 (97.9)	1361 (97.6)	1289 (91.9)	2622 (91.6)	< 0.001
	Yes	449 (5.3)	61 (2.1)	33 (2.4)	114 (8.1)	241 (8.4)	
BMI (mean (SD))		23.97 (3.70)	22.39 (3.97)	22.98 (3.64)	24.08 (3.52)	24.58 (3.87)	< 0.001
SBP (mean (SD))		128.52 (20.22)	125.76 (19.72)	127.02 (19.88)	128.77 (20.36)	131.91 (20.32)	< 0.001
TyG (mean (SD))		8.65 (0.64)	8.11 (0.30)	8.26 (0.23)	8.99 (0.39)	9.21 (0.54)	< 0.001
Remnant cholesterol (mean (SD))		24.79 (23.78)	11.88 (7.90)	14.81 (9.46)	28.87 (15.31)	40.67 (31.47)	< 0.001
Non-HDL cholesterol (mean (SD))		141.83 (37.65)	109.95 (18.88)	161.91 (21.08)	118.21 (16.30)	175.73 (29.71)	< 0.001
CRP (mean (SD))		2.76 (7.55)	2.99 (8.80)	2.40 (6.49)	2.95 (8.21)	2.60 (6.21)	0.048
HBA1c (mean (SD))		5.27 (0.80)	5.08 (0.44)	5.16 (0.50)	5.34 (0.93)	5.47 (1.04)	< 0.001

**Table 2:** The association between TyG, non-HDL cholesterol, remnant cholesterol and their joint effects with incident stroke risk in CHARLs.

		Events/Sample	Incidence rate^a^	Model 1^b^	*p*-value	Model 2^c^	*p*-value
TyG	Below median (Reference)	219/4278	6.38	1.00		1.00	
	Above median	333/4266	9.87	1.59 (1.34–1.88)	< 0.001	1.41 (1.18–1.69)	< 0.001
Per 1-SD				1.27 (1.18–1.37)	< 0.001	1.20 (1.11–1.30)	< 0.001
Non-HDL cholesterol	Below median (Reference)	215/4287	6.33	1.00		1.00	
	Above median	337/4257	9.88	1.57 (1.32–1.86)	< 0.001	1.49 (1.25–1.78)	< 0.001
Per 1-SD				1.23 (1.14–1.33)		1.19 (1.10–1.29)	< 0.001
Remnant cholesterol	Below median (Reference)	230/4172	6.90	1.00		1.00	
	Above median	322/4372	9.28	1.37 (1.15–1.62)	< 0.001	1.26 (1.06–1.50)	0.01
Per 1-SD				1.14 (1.07–1.22)	< 0.001	1.11 (1.03–1.18)	0.004
TyG and non-HDL cholesterol	Both below median (Reference)	129/2884	5.62	1.00		1.00	
	Only TyG above median	86/1403	7.83	1.44 (1.09–1.89)	0.01	1.33 (1.04–1.79)	0.02
	Only Non-HDL cholesterol above median	90/1394	7.94	1.41 (1.08–1.985)	0.01	1.44 (1.10–1.89)	0.01
	Both above median	247/2863	10.85	1.98 (1.60–2.45)	< 0.001	1.76 (1.41–2.20)	< 0.001
TyG and Remnant cholesterol	Both below median (Reference)	169/3364	6.25	1.00		1.00	
	Only TyG above median	61/808	9.69	1.60 (1.20–2.15)	0.002	1.38 (1.05–1.76)	0.02
	Only remnant cholesterol above median	50/914	6.92	1.11 (0.81–1.52)	0.52	1.07 (0.77–1.47)	0.66
	Both above median	272/3458	9.91	1.63 (1.34–1.97)	< 0.001	1.45 (1.18–1.77)	< 0.001

a: Incidence rates were reported as per 1000 person-years.

b: Unadjusted models.

c. Adjusted models with age, sex, education, marital status, residency, smoking and drinking status, BMI, SBP, C-reactive protein and HbA1c.

### Association of TyG, Non-HDL cholesterol, remnant cholesterol and their joint effects with incident stroke risk

**Fig. 2:**
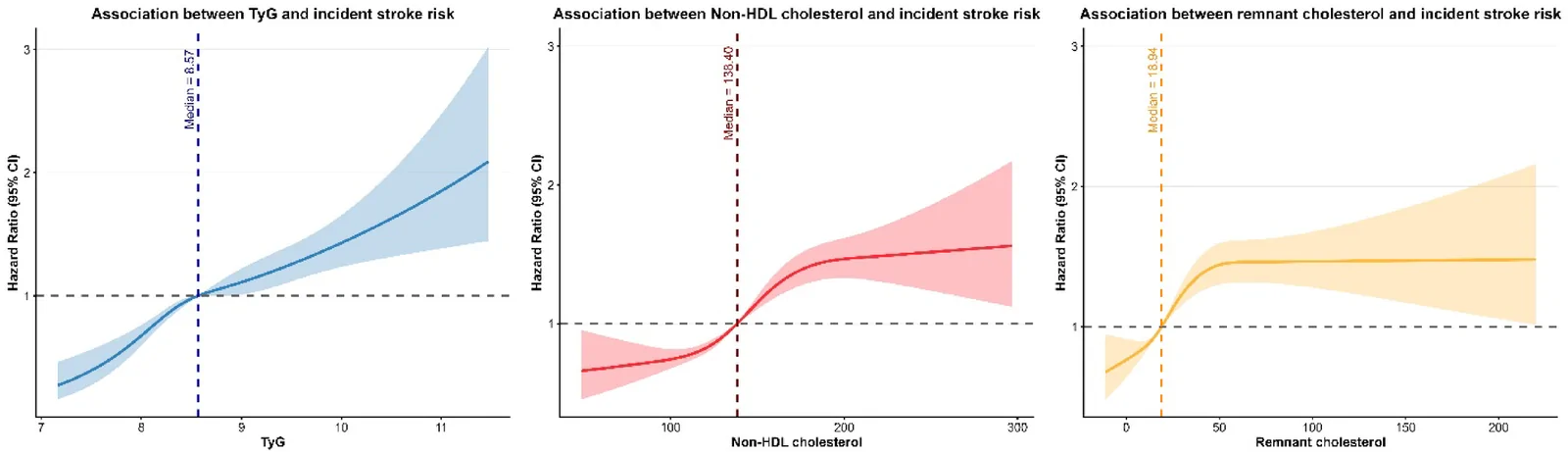
Restricted cubic splines of the non-linear association between TyG, non-HDL cholesterol, remnant cholesterol and stroke risk.

Table 2 summarizes the associations of the TyG index, atherogenic cholesterol markers, and their joint categories with incident stroke based on Cox proportional hazards models, and Fig. 2 illustrates the corresponding dose–response relationships using restricted cubic splines. In the fully adjusted models, participants with TyG, non–HDL cholesterol, or remnant cholesterol values at or above the median had significantly higher risks of incident stroke compared with those below the median (HR for TyG: 1.41; 95% CI 1.18–1.69; HR for non–HDL cholesterol: 1.49; 95% CI 1.25–1.78; HR for remnant cholesterol: 1.26; 95% CI 1.06–1.50). Spline analyses provided evidence of significant but nonlinear associations between each biomarker and incident stroke (p for nonlinearity: TyG = 0.02; non–HDL cholesterol < 0.001; remnant cholesterol < 0.001).

In the joint analyses of the TyG index with atherogenic cholesterol markers, participants with both biomarkers at or above the median had the highest risk of incident stroke (Table 2). Compared with those with both TyG and non–HDL cholesterol below the median, individuals with both markers at or above the median had an HR of 1.76 (95% CI: 1.41–2.20) in the fully adjusted model. Similarly, participants with both TyG and remnant cholesterol at or above the median had an HR of 1.45 (95% CI: 1.18–1.77) compared with the corresponding reference group.

### The predictive value of incidence stroke risk with TyG and atherogenic cholesterol markers

Results of the ROC curves were reported in Supplementary Fig. 1. The reference model including traditional risk factors alone had an AUC of 0.564 (95% CI: 0.554–0.573), indicating limited discrimination. Adding each biomarker individually modestly improved discrimination, with AUCs increasing to 0.648 (95% CI: 0.641–0.654) for the model including TyG, 0.642 (95% CI: 0.635–0.647) for the model including remnant cholesterol, and 0.652 (95% CI: 0.646–0.656) for the model including non-HDL cholesterol.

When two biomarkers were added jointly, the AUC was 0.653 (95% CI: 0.646–0.658) for the model including TyG and remnant cholesterol, and 0.661 (95% CI: 0.654–0.666) for the model including TyG and non-HDL cholesterol. Although the combination of TyG and non-HDL cholesterol showed the highest AUC among the evaluated models, the overall discriminatory performance remained modest.

### Stratified analysis and sensitivity analysis

**Fig. 3:**
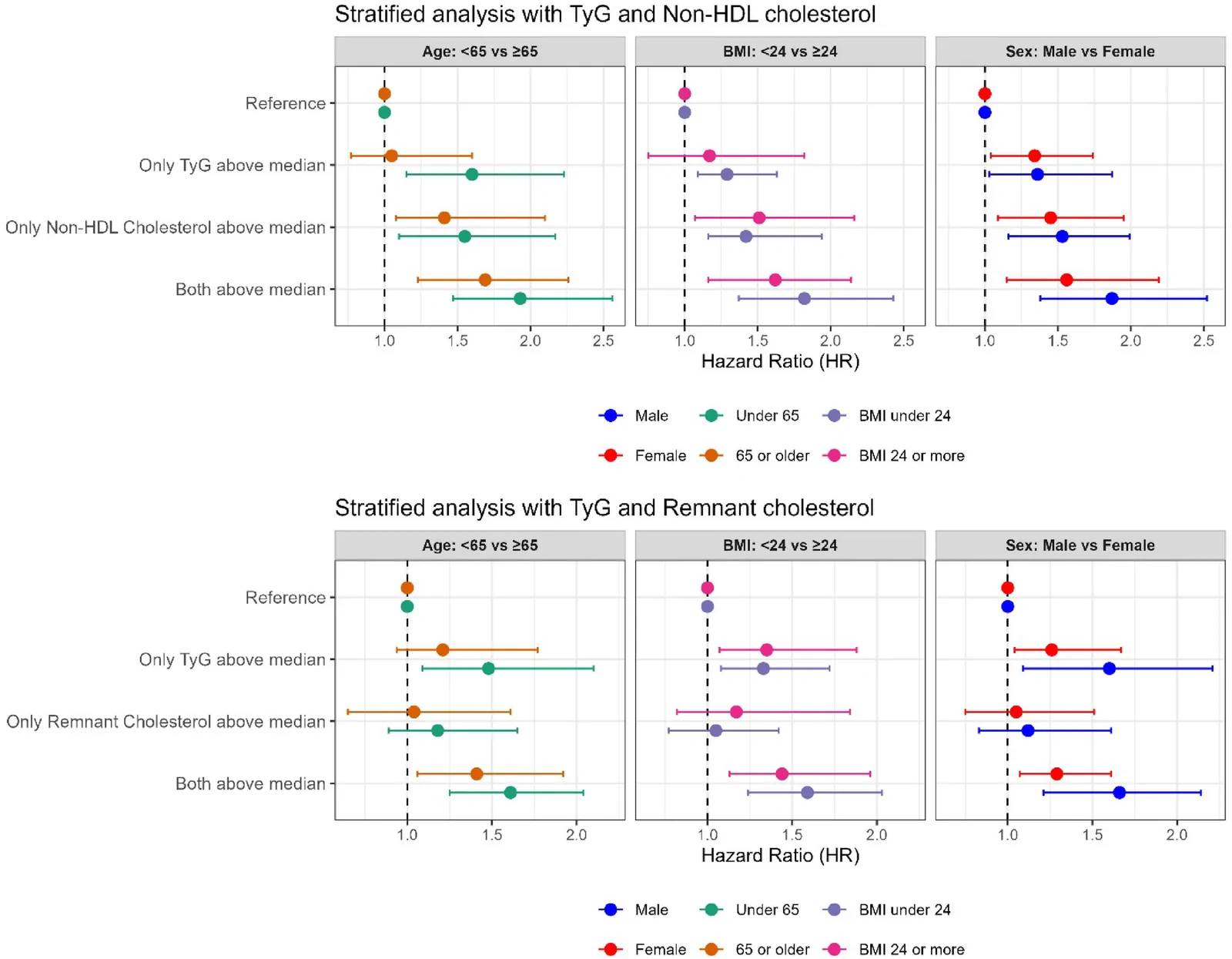
Stratified analysis of TyG and non-HDL cholesterol/remnant cholesterol with stroke risk by age, sex, and BMI.

Stratified analyses by age, BMI, and sex are presented in Fig. 3. Overall, the patterns of association between the joint categories of the TyG index and atherogenic cholesterol markers and incident stroke were broadly consistent across subgroups, with no evidence of interaction (Supplementary Tables 6–7). In all strata, participants with both TyG and non–HDL cholesterol at or above the median, or with both TyG and remnant cholesterol at or above the median, had the highest risk compared with those with both biomarkers below the median. Elevated TyG alone or elevated non–HDL cholesterol alone was also associated with increased stroke risk in most subgroups while elevated remnant cholesterol alone did not show significant higher risk. The magnitude of association tended to be slightly greater among participants who were under 65, who were not obese (BMI < 24 kg/m^2^), and who were males. Detailed results of the subgroup analysis were provided in Supplementary Tables 6–7.

The results were consistent across multiple sensitivity analyses, including complete-case analysis, exclusion of participants who developed incident stroke within the first two years of follow-up, and additional adjustment for medication use, supporting the robustness of our findings (Supplementary Table 10). In sensitivity analysis of accounting death as a competing event using Fine–Gray competing risk regression models, the results remained largely consistent with those from the primary Cox regression analyses (Supplementary Table 11). Participants who had both elevated TyG and elevated atherogenic lipid markers continuing to show the highest risk of incident stroke. In addition, when the exposures were reclassified into tertiles, the joint associations of TyG and atherogenic lipid markers with incident stroke showed graded patterns (Supplementary Tables 12–14).

## Discussion

In this large, prospective cohort of middle-aged and older Chinese adults, we systematically examined the independent and joint associations of the TyG index and atherogenic cholesterol markers with incident stroke risk. We found that higher TyG levels and higher atherogenic cholesterol levels were each associated with an increased risk of stroke, with the greatest risk observed among participants in the highest categories of both TyG and either non-HDL cholesterol or remnant cholesterol. In addition, incorporating the TyG index and cholesterol markers into a baseline model containing traditional cardiovascular risk factors modestly improved the discriminative ability for stroke. Overall, our findings highlight the importance of considering both metabolic dysfunction and atherogenic dyslipidemia when evaluating stroke risk in middle-aged and older adults.

The cumulative incidence of stroke observed in our study was generally consistent with previous evidence from similar middle-aged and older Chinese populations. In the present cohort, 552 of 8,544 participants developed incident stroke during follow-up, corresponding to a crude cumulative incidence of 6.5% over a maximum follow-up of 9 years. Previous studies have reported broadly comparable estimates, although with some variation. For example, a national cohort study of cardiometabolic index and incident stroke reported 418 stroke events among 7,821 participants, corresponding to a cumulative incidence of 5.3% over a mean follow-up of 7 years^18^. Another CHARLS-based study reported 457 incident stroke cases among 7,455 participants over 7 years of follow-up, corresponding to an incidence of 6.13%^19^.

Consistent with prior research, our findings support the association of insulin resistance-related and atherogenic lipid markers with stroke and cardiovascular risk. A meta-analysis of cohort studies reported that higher TyG index was associated with increased stroke risk, supporting the role of insulin resistance in cerebrovascular disease^20^. Prior prospective cohort studies have also linked TyG-related composite indices and other cardiometabolic markers with incident stroke in middle-aged and older Chinese adults^21,22^. In parallel, previous studies have suggested that non-HDL cholesterol and remnant cholesterol may better reflect atherogenic lipoprotein burden and residual vascular risk than LDL cholesterol alone^23,24^. For example, data from the Copenhagen General Population Study suggested that the proportion of ischemic stroke attributable to elevated non-HDL cholesterol was approximately twice that attributable to elevated LDL cholesterol^25^. Higher non-HDL cholesterol levels have also been associated with increased risks of recurrent stroke, intracranial hemorrhage, and all-cause mortality among patients with acute stroke^26^. Taken together, this body of evidence reinforces our finding that the TyG index and atherogenic cholesterol markers are independent and significant predictors of stroke risk.

Our study extends beyond current literature by jointly assessing the effects of the TyG index and atherogenic cholesterol markers on incident stroke risk. We further demonstrated that combining the TyG index with non-HDL cholesterol yielded modestly improved discrimination for stroke risk than either biomarker alone, underscoring the added value of integrating complementary metabolic and lipid markers in risk stratification. This joint assessment is advantageous because it captures both insulin resistance–related dysregulation and atherogenic lipoprotein burden, thereby more fully reflecting the underlying pathophysiology that predisposes to cerebrovascular events. Mechanistically, a higher TyG index reflects systemic insulin resistance, which is closely linked to endothelial dysfunction, low-grade inflammation, oxidative stress, and prothrombotic states, all of which promote cerebrovascular damage^27–29^. In parallel, elevated non-HDL cholesterol and remnant cholesterol represent increased concentrations of atherogenic apoB-containing lipoproteins that can penetrate the arterial wall, accelerate atherosclerotic plaque formation, and contribute to plaque instability and thrombosis^23,30,31^. The combination of these pathways may therefore synergistically amplify stroke risk, consistent with the stronger associations observed in participants with simultaneously elevated TyG and atherogenic cholesterol markers.

Collectively, these findings have several implications for public health and clinical practice. First, our study suggests that simultaneous consideration of insulin resistance and atherogenic dyslipidemia may improve risk classification for stroke beyond traditional factors alone, particularly among middle-aged and older adults with higher risk of stroke. Because the TyG index and non-HDL cholesterol or remnant cholesterol can be derived from routinely available fasting glucose and lipid profiles, incorporating these markers into primary care–based screening or existing risk prediction tools could provide a low-cost, scalable strategy for early identification of individuals at heightened cerebrovascular risk. In clinical practice, patients with elevated TyG and non-HDL cholesterol might warrant closer monitoring, earlier initiation or intensification of lipid-lowering therapy, and comprehensive management of insulin resistance through lifestyle and pharmacological interventional strategies. From a public health perspective, our results reinforce the need for integrated prevention programs that jointly target risk factors, particularly insulin resistance, dyslipidemia, rather than addressing these risks in isolation. Such programs may be especially relevant in settings with a high burden of stroke and rapidly rising prevalence of diabetes and obesity, where simple composite markers like TyG and non-HDL cholesterol could help prioritize limited resources toward those at greatest risk. 

Several limitations should be considered when interpreting the results of our findings. First, as an observational cohort study, our findings cannot establish causality, and despite extensive adjustment for potential confounders, the possibility of residual or unmeasured confounding (lifestyle information, unmeasured/unreported comorbidities) cannot be excluded. Residual confounding related to post-baseline treatment therefore cannot be excluded. Second, stroke events were ascertained using self-reported physician diagnosis during follow-up interviews, as registry-confirmed or medically adjudicated stroke outcomes were not available in CHARLS. This approach may introduce recall bias, underreporting, and outcome misclassification. We also lacked detailed information on stroke subtype, severity, imaging confirmation, hospitalization, and acute treatment, which precluded separate analyses of ischemic and hemorrhagic stroke^32^. Third, we only assessed TyG and atherogenic cholesterol markers at baseline and did not account for changes in these risk factors over time. The variability, cumulative exposure, or trajectories of these markers may influence stroke risk and should be explored in future studies with repeated measurements^33^. Fourth, attrition due to loss to follow-up may have introduced selection bias if participants who were lost differed systematically in their metabolic or vascular risk profiles from those who remained in the cohort. Fifth, our sample consisted of Chinese adults aged ≥ 45 years, so caution is warranted in generalizing these results to younger individuals or to other ethnic groups with different baseline risk factor patterns and healthcare contexts. Finally, although we assessed the incremental discriminatory value of TyG and atherogenic lipid markers beyond a traditional risk-factor model, we did not directly compare these biomarkers with established clinical risk prediction scores. Future studies should examine whether incorporating TyG, and atherogenic lipid biomarkers into population-specific risk prediction models improves calibration, reclassification, and clinical utility for stroke prevention.

## Conclusion

In this large prospective cohort of middle-aged and older Chinese adults, higher TyG index and atherogenic cholesterol markers were both independently and jointly associated with an increased risk of stroke. Adding TyG and atherogenic cholesterol markers to traditional risk factors modestly improved stroke risk prediction, suggesting their potential utility for more refined risk stratification. These findings contribute to the growing evidence linking metabolic dysfunction and atherogenic dyslipidemia to cerebrovascular disease and provide a rationale for future studies and prevention strategies that concurrently target both pathways to reduce stroke burden.

## Supplementary Information

Below is the link to the electronic supplementary material.

Supplementary Material 1

## Data Availability

The datasets presented in this study can be found in online repositories. These data can be found here: (http://charls.pku.edu.cn/).
