# Supplementary material for: Joint association of triglyceride-glucose index and atherogenic lipid markers with incident stroke risk
Source: Sci Rep. 2026 Jun 12;16:26858. doi: 10.1038/s41598-026-56940-5 (PMC13518814; doi:10.1038/s41598-026-56940-5)
Supplement: Supplementary file 1 [file 41598_2026_56940_MOESM1_ESM.docx]

Supplementary Figure 1: ROC curve of TyG and atherogenic cholesterol markers for incident stroke risk discrimination in CHARLS.

Supplementary Table 1. Baseline characteristics of participants based on TyG and remnant cholesterol status.

|  | **level** | **Overall** | **Reference group** | **Only RC above median** | **Only TyG above median** | **Both above median** | **p** |
| --- | --- | --- | --- | --- | --- | --- | --- |
| N |  | 8544 | 3364 | 914 | 808 | 3458 |  |
| Age (mean (SD)) |  | 59.03 (9.45) | 59.09 (9.59) | 59.25 (9.95) | 60.54 (9.46) | 58.56 (9.15) | <0.001 |
| Sex (%) | Female | 4448 (52.1) | 1622 (48.2) | 430 (47.0) | 475 (58.8) | 1921 (55.6) | <0.001 |
|  | Male | 4096 (47.9) | 1742 (51.8) | 484 (53.0) | 333 (41.2) | 1537 (44.4) |  |
| Education (%) | Below High School | 7648 (89.5) | 3033 (90.2) | 825 (90.3) | 732 (90.6) | 3058 (88.4) | 0.219 |
|  | College or above | 108 (1.3) | 36 (1.1) | 10 (1.1) | 10 (1.2) | 52 (1.5) |  |
|  | High school | 788 (9.2) | 295 (8.8) | 79 (8.6) | 66 (8.2) | 348 (10.1) |  |
| Marry (%) | Marry | 7546 (88.3) | 2974 (88.4) | 813 (88.9) | 699 (86.5) | 3060 (88.5) | 0.386 |
|  | Other | 998 (11.7) | 390 (11.6) | 101 (11.1) | 109 (13.5) | 398 (11.5) |  |
| Residency (%) | Rural | 5466 (64.0) | 2301 (68.4) | 609 (66.6) | 529 (65.5) | 2027 (58.6) | <0.001 |
|  | Urban | 3078 (36.0) | 1063 (31.6) | 305 (33.4) | 279 (34.5) | 1431 (41.4) |  |
| Current smoker (%) | No | 5958 (69.7) | 2297 (68.3) | 570 (62.4) | 607 (75.1) | 2484 (71.8) | <0.001 |
|  | Yes | 2586 (30.3) | 1067 (31.7) | 344 (37.6) | 201 (24.9) | 974 (28.2) |  |
| Current drinker (%) | No | 5576 (65.3) | 2115 (62.9) | 583 (63.8) | 578 (71.5) | 2300 (66.5) | <0.001 |
|  | Yes | 2968 (34.7) | 1249 (37.1) | 331 (36.2) | 230 (28.5) | 1158 (33.5) |  |
| Hypertension (%) | No | 6553 (76.7) | 2780 (82.6) | 746 (81.6) | 582 (72.0) | 2445 (70.7) | <0.001 |
|  | Yes | 1991 (23.3) | 584 (17.4) | 168 (18.4) | 226 (28.0) | 1013 (29.3) |  |
| Dyslipidemia (%) | No | 7843 (91.8) | 3201 (95.2) | 863 (94.4) | 728 (90.1) | 3051 (88.2) | <0.001 |
|  | Yes | 701 (8.2) | 163 (4.8) | 51 (5.6) | 80 (9.9) | 407 (11.8) |  |
| Diabetes (%) | No | 8095 (94.7) | 3295 (97.9) | 889 (97.3) | 713 (88.2) | 3198 (92.5) | <0.001 |
|  | Yes | 449 (5.3) | 69 (2.1) | 25 (2.7) | 95 (11.8) | 260 (7.5) |  |
| BMI (mean (SD)) |  | 23.97 (3.70) | 22.56 (3.85) | 22.72 (3.96) | 23.21 (3.88) | 24.95 (3.51) | <0.001 |
| SBP (mean (SD)) |  | 128.52 (20.22) | 125.99 (19.46) | 126.82 (20.89) | 131.57 (21.82) | 130.72 (20.03) | <0.001 |
| TyG (mean (SD)) |  | 8.65 (0.64) | 8.12 (0.29) | 8.30 (0.24) | 8.80 (0.24) | 9.22 (0.52) | <0.001 |
| Remnant cholesterol (mean (SD)) |  | 24.79 (23.78) | 9.55 (5.58) | 24.93 (6.45) | 13.64 (4.45) | 42.19 (28.18) | <0.001 |
| Non-HDL cholesterol (mean (SD)) |  | 141.83 (37.65) | 124.70 (31.00) | 134.91 (31.01) | 146.41 (34.61) | 159.25 (37.80) | <0.001 |
| CRP (mean (SD)) |  | 2.76 (7.55) | 2.72 (7.80) | 3.10 (9.22) | 2.87 (8.88) | 2.68 (6.39) | 0.469 |
| HBA1c (mean (SD)) |  | 5.27 (0.80) | 5.11 (0.45) | 5.11 (0.51) | 5.52 (1.17) | 5.41 (0.97) | <0.001 |

Supplementary Table 2. Missing rates (%) of covariates overall and by TyG and Non-HDL cholesterol groups.

| **Covariate** | **Overall missing rate%** | **Both above median** | **Only Non HDL-C above median** | **Only TyG above median** | **Reference group** |
| --- | --- | --- | --- | --- | --- |
| Sex | 0.00 | 0.00 | 0.00 | 0.00 | 0.00 |
| Hypertension | 0.51 | 0.40 | 0.38 | 0.85 | 0.54 |
| Dyslipidemia | 2.01 | 2.35 | 1.34 | 2.44 | 1.82 |
| Diabetes | 0.88 | 1.10 | 0.86 | 0.85 | 0.69 |
| Residency | 0.00 | 0.00 | 0.00 | 0.00 | 0.00 |
| Marry | 0.00 | 0.00 | 0.00 | 0.00 | 0.00 |
| Age | 0.00 | 0.00 | 0.00 | 0.00 | 0.00 |
| Education | 0.00 | 0.00 | 0.00 | 0.00 | 0.00 |
| Current drinker | 1.58 | 1.10 | 1.43 | 2.33 | 1.78 |
| Current smoker | 0.03 | 0.00 | 0.00 | 0.00 | 0.10 |
| BMI | 12.75 | 11.84 | 12.79 | 15.78 | 12.23 |
| SBP | 12.57 | 12.14 | 12.79 | 15.15 | 11.69 |
| CRP | 0.00 | 0.00 | 0.00 | 0.00 | 0.00 |
| HbA1c | 0.66 | 0.40 | 1.15 | 0.53 | 0.74 |

Supplementary Table 3. Missing rate of covariates overall and by TyG and Non-HDL cholesterol groups.

| **Variable** | **Overall missing rate %** | **Both above median** | **Only RC above median** | **Only TyG above median** | **Reference group** |
| --- | --- | --- | --- | --- | --- |
| Sex | 0.00 | 0.00 | 0.00 | 0.00 | 0.00 |
| Hypertension | 0.51 | 0.54 | 0.00 | 0.56 | 0.62 |
| Dyslipidemia | 2.10 | 2.03 | 0.47 | 3.91 | 1.97 |
| Diabetes | 0.88 | 0.87 | 0.47 | 1.68 | 0.82 |
| Residency | 0.00 | 0.00 | 0.00 | 0.00 | 0.00 |
| Marry | 0.00 | 0.00 | 0.00 | 0.00 | 0.00 |
| Age | 0.00 | 0.00 | 0.00 | 0.00 | 0.00 |
| Education | 0.00 | 0.00 | 0.00 | 0.00 | 0.00 |
| Current smoker | 1.58 | 1.54 | 1.56 | 1.30 | 1.69 |
| Current drinker | 0.03 | 0.00 | 0.00 | 0.00 | 0.08 |
| BMI | 12.75 | 13.12 | 12.60 | 13.04 | 12.37 |
| SBP | 12.57 | 13.16 | 11.82 | 12.85 | 12.12 |
| CRP | 0.00 | 0.00 | 0.00 | 0.00 | 0.00 |
| HbA1c | 0.66 | 0.46 | 0.31 | 0.37 | 1.03 |

Supplementary Table 4. Baseline characteristics of participants based on TyG and remnant cholesterol status without multiple imputation.

|  | **Level** | **Overall** | **Reference group** | **Only Non HDL-C above median** | **Only TyG above median** | **Both above median** | **p** |
| --- | --- | --- | --- | --- | --- | --- | --- |
| N |  | 8544 | 2884 | 1394 | 1403 | 2863 |  |
| Age (mean (SD)) |  | 59.03 (9.45) | 59.08 (9.80) | 59.23 (9.37) | 58.73 (9.36) | 59.04 (9.18) | 0.551 |
| Sex (%) | Female | 4448 (52.1) | 1310 (45.4) | 742 (53.2) | 723 (51.5) | 1673 (58.4) | <0.001 |
|  | Male | 4096 (47.9) | 1574 (54.6) | 652 (46.8) | 680 (48.5) | 1190 (41.6) |  |
| Education (%) | Below High School | 7648 (89.5) | 2607 (90.4) | 1251 (89.7) | 1240 (88.4) | 2550 (89.1) | 0.274 |
|  | College or above | 108 (1.3) | 34 (1.2) | 12 (0.9) | 23 (1.6) | 39 (1.4) |  |
|  | High school | 788 (9.2) | 243 (8.4) | 131 (9.4) | 140 (10.0) | 274 (9.6) |  |
| Marry (%) | Marry | 7546 (88.3) | 2551 (88.5) | 1236 (88.7) | 1229 (87.6) | 2530 (88.4) | 0.819 |
|  | Other | 998 (11.7) | 333 (11.5) | 158 (11.3) | 174 (12.4) | 333 (11.6) |  |
| Residency (%) | Rural | 5466 (64.0) | 1995 (69.2) | 915 (65.6) | 866 (61.7) | 1690 (59.0) | <0.001 |
|  | Urban | 3078 (36.0) | 889 (30.8) | 479 (34.4) | 537 (38.3) | 1173 (41.0) |  |
| Current smoker (%) | No | 5836 (69.7) | 1839 (65.1) | 966 (70.9) | 945 (69.1) | 2086 (74.0) | <0.001 |
|  | Yes | 2538 (30.3) | 986 (34.9) | 396 (29.1) | 422 (30.9) | 734 (26.0) |  |
| Current drinker (%) | No | 5574 (65.3) | 1780 (61.8) | 917 (65.8) | 917 (65.4) | 1960 (68.5) | <0.001 |
|  | Yes | 2965 (34.7) | 1101 (38.2) | 476 (34.2) | 485 (34.6) | 903 (31.5) |  |
| Hypertension (%) | No | 6526 (76.7) | 2377 (82.8) | 1135 (81.7) | 1028 (73.9) | 1986 (69.7) | <0.001 |
|  | Yes | 1977 (23.3) | 493 (17.2) | 255 (18.3) | 364 (26.1) | 865 (30.3) |  |
| Dyslipidemia (%) | No | 7688 (91.8) | 2703 (95.4) | 1294 (94.3) | 1256 (91.7) | 2435 (86.9) | <0.001 |
|  | Yes | 687 (8.2) | 130 (4.6) | 78 (5.7) | 113 (8.3) | 366 (13.1) |  |
| Diabetes (%) | No | 8027 (94.7) | 2804 (97.9) | 1351 (97.6) | 1277 (91.9) | 2595 (91.5) | <0.001 |
|  | Yes | 447 (5.3) | 61 (2.1) | 33 (2.4) | 113 (8.1) | 240 (8.5) |  |
| BMI (mean (SD)) |  | 23.97 (3.70) | 22.39 (3.97) | 22.98 (3.64) | 27.08 (3.52) | 24.58 (3.87) | <0.001 |
| SBP (mean (SD)) |  | 128.81 (21.03) | 125.53 (20.41) | 127.10 (20.76) | 129.52 (21.40) | 132.59 (20.98) | <0.001 |
| TyG (mean (SD)) |  | 8.65 (0.64) | 8.11 (0.30) | 8.26 (0.23) | 8.99 (0.39) | 9.21 (0.54) | <0.001 |
| Remnant cholesterol (mean (SD)) |  | 24.79 (23.78) | 11.88 (7.90) | 14.81 (9.46) | 28.87 (15.31) | 40.67 (31.47) | <0.001 |
| Non-HDL cholesterol (mean (SD)) |  | 141.83 (37.65) | 109.95 (18.88) | 161.91 (21.08) | 118.21 (16.30) | 175.73 (29.71) | <0.001 |
| CRP (mean (SD)) |  | 2.76 (7.55) | 2.99 (8.80) | 2.40 (6.49) | 2.95 (8.21) | 2.60 (6.21) | 0.048 |
| HBA1c (mean (SD)) |  | 5.27 (0.80) | 5.08 (0.44) | 5.17 (0.50) | 5.34 (0.93) | 5.47 (1.05) | <0.001 |

Supplementary Table 5. Baseline characteristics of participants based on TyG and remnant cholesterol status without multiple imputation.

|  | **Level** | **Overall** | **Reference group** | **Only RC above median** | **Only TyG above median** | **Both above median** | **p** |
| --- | --- | --- | --- | --- | --- | --- | --- |
| N |  | 8544 | 3364 | 914 | 808 | 3458 |  |
| Age (mean (SD)) |  | 59.03 (9.45) | 59.09 (9.59) | 59.25 (9.95) | 60.54 (9.46) | 58.56 (9.15) | <0.001 |
| Sex (%) | Female | 4448 (52.1) | 1622 (48.2) | 430 (47.0) | 475 (58.8) | 1921 (55.6) | <0.001 |
|  | Male | 4096 (47.9) | 1742 (51.8) | 484 (53.0) | 333 (41.2) | 1537 (44.4) |  |
| Education (%) | Below High School | 7648 (89.5) | 3033 (90.2) | 825 (90.3) | 732 (90.6) | 3058 (88.4) | 0.219 |
|  | College or above | 108 (1.3) | 36 (1.1) | 10 (1.1) | 10 (1.2) | 52 (1.5) |  |
|  | High school | 788 (9.2) | 295 (8.8) | 79 (8.6) | 66 (8.2) | 348 (10.1) |  |
| Marry (%) | Marry | 7546 (88.3) | 2974 (88.4) | 813 (88.9) | 699 (86.5) | 3060 (88.5) | 0.386 |
|  | Other | 998 (11.7) | 390 (11.6) | 101 (11.1) | 109 (13.5) | 398 (11.5) |  |
| Residency (%) | Rural | 5466 (64.0) | 2301 (68.4) | 609 (66.6) | 529 (65.5) | 2027 (58.6) | <0.001 |
|  | Urban | 3078 (36.0) | 1063 (31.6) | 305 (33.4) | 279 (34.5) | 1431 (41.4) |  |
| Current smoker (%) | No | 5836 (69.7) | 2253 (68.4) | 552 (62.0) | 595 (74.9) | 2436 (71.8) | <0.001 |
|  | Yes | 2538 (30.3) | 1043 (31.6) | 339 (38.0) | 199 (25.1) | 957 (28.2) |  |
| Current drinker (%) | No | 5574 (65.3) | 2114 (62.9) | 583 (63.8) | 577 (71.5) | 2300 (66.5) | <0.001 |
|  | Yes | 2965 (34.7) | 1246 (37.1) | 331 (36.2) | 230 (28.5) | 1158 (33.5) |  |
| Hypertension (%) | No | 6526 (76.7) | 2766 (82.7) | 746 (81.6) | 580 (72.3) | 2434 (70.7) | <0.001 |
|  | Yes | 1977 (23.3) | 580 (17.3) | 168 (18.4) | 222 (27.7) | 1007 (29.3) |  |
| Dyslipidemia (%) | No | 7688 (91.8) | 3139 (95.2) | 858 (94.6) | 704 (90.1) | 2987 (88.1) | <0.001 |
|  | Yes | 687 (8.2) | 159 (4.8) | 49 (5.4) | 77 (9.9) | 402 (11.9) |  |
| Diabetes (%) | No | 8027 (94.7) | 3269 (97.9) | 886 (97.3) | 700 (88.1) | 3172 (92.5) | <0.001 |
|  | Yes | 447 (5.3) | 69 (2.1) | 25 (2.7) | 95 (11.9) | 258 (7.5) |  |
| BMI (mean (SD)) |  | 23.97 (3.70) | 22.55 (3.83) | 22.70 (3.97) | 27.13 (3.85) | 24.96 (3.58) | <0.001 |
| SBP (mean (SD)) |  | 128.81 (21.03) | 125.83 (20.21) | 126.80 (21.71) | 132.31 (22.72) | 131.43 (20.77) | <0.001 |
| TyG (mean (SD)) |  | 8.65 (0.64) | 8.12 (0.29) | 8.30 (0.24) | 8.80 (0.24) | 9.22 (0.52) | <0.001 |
| Remnant cholesterol (mean (SD)) |  | 24.79 (23.78) | 9.55 (5.58) | 24.93 (6.45) | 13.64 (4.45) | 42.19 (28.18) | <0.001 |
| Non-HDL cholesterol (mean (SD)) |  | 141.83 (37.65) | 124.70 (31.00) | 134.91 (31.01) | 146.41 (34.61) | 159.25 (37.80) | <0.001 |
| CRP (mean (SD)) |  | 2.76 (7.55) | 2.72 (7.80) | 3.10 (9.22) | 2.87 (8.88) | 2.68 (6.39) | 0.469 |
| HBA1c (mean (SD)) |  | 5.27 (0.80) | 5.11 (0.45) | 5.11 (0.51) | 5.52 (1.18) | 5.41 (0.97) | <0.001 |

Supplementary Table 6: Subgroup analysis of the joint association of TyG and Non-HDL cholesterol with incident stroke risk in CHARLS.

|  |  | Both below median | Only TyG above median | Only Non-HDL above median | Both above median |
| --- | --- | --- | --- | --- | --- |
| By sex P for interaction=0.49 | |  |  |  |  |
| Male | Events/Sample (Incidence rate) | 75/1574 (6.13) | 51/680 (8.47) | 44/652 (9.82) | 115/1190 (12.49) |
|  | HR | 1.00 (Ref) | 1.36 (1.03-1.87) | 1.53 (1.16-1.99) | 1.87 (1.38-2.52) |
| Female | Events/Sample (Incidence rate) | 54/1310 (5.03) | 35/723 (6.05) | 46/742 (7.50) | 132/1673 (9.73) |
|  | HR | 1.00 (Ref) | 1.34 (1.04-1.74) | 1.45 (1.09-1.95) | 1.56 (1.15-2.19) |
| By age group P for interaction=0.29 | |  |  |  |  |
| <65 | Events/Sample (Incidence rate) | 77/2085 (4.43) | 66/1058 (6.91) | 59/1018 (7.64) | 173/2146 (9.78) |
|  | HR | 1.00 (Ref) | 1.60 (1.15-2.19) | 1.55 (1.10-2.17) | 1.93 (1.47-2.56) |
| 65+ | Events/Sample (Incidence rate) | 52/799 (8.51) | 20/345 (9.28) | 32/376 (11.09) | 74/717 (14.54) |
|  | HR | 1.00 (Ref) | 1.05 (0.77-1.60) | 1.41 (1.08-2.10) | 1.69 (1.23-2.26) |
| By BMI levels P for interaction=0.51 | |  |  |  |  |
| BMI<24 | Events/Sample (Incidence rate) | 89/2179 (5.15) | 51/946 (6.62) | 47/843 (7.22) | 107/1431 (9.52) |
|  | HR | 1.00 (Ref) | 1.29 (1.09-1.63) | 1.42 (1.16-1.94) | 1.82 (1.37-2.43) |
| BMI>=24 | Events/Sample (Incidence rate) | 40/75 (7.03) | 39/560 (8.71) | 39/448 (10.75) | 140/1431 (12.41) |
|  | HR | 1.00 (Ref) | 1.17 (0.75-1.82) | 1.51 (1.07-2.16) | 1.62 (1.16-2.14) |

Supplementary Table 7: Subgroup analysis of the joint association of TyG and remnant cholesterol with incident stroke risk in CHARLS.

|  |  | Both below median | Only TyG above median | Only RC above median | Both above median |
| --- | --- | --- | --- | --- | --- |
| By sex P for interaction=0.27 | |  |  |  |  |
| Male | Events/Sample (Incidence rate) | 91/1742 (6.66) | 29/333 (10.59) | 28/484 (7.44) | 137/1537 (11.51) |
|  | HR | 1.00 (Ref) | 1.60 (1.09-2.21) | 1.12 (0.83-1.61) | 1.66 (1.21-2.14) |
| Female | Events/Sample (Incidence rate) | 78/1622 (5.82) | 32/475 (8.42) | 22/430 (6.32) | 135/1921 (9.68) |
|  | HR | 1.00 (Ref) | 1.26 (1.04-1.67) | 1.05 (0.75-1.51) | 1.29 (1.07-1.61) |
| By age group P for interaction=0.42 | |  |  |  |  |
| <65 | Events/Sample (Incidence rate) | 103/2441 (5.04) | 39/556 (8.63) | 33/662 (6.02) | 200/2648 (9.17) |
|  | HR | 1.00 (Ref) | 1.48 (1.09-2.10) | 1.18 (0.89-1.65) | 1.61 (1.25-2.04) |
| 65+ | Events/Sample (Incidence rate) | 66/923 (9.62) | 22/252 (11.35) | 17/252 (9.95) | 72/810 (12.73) |
|  | HR | 1.00 (Ref) | 1.21 (0.94-1.77) | 1.04 (0.65-1.61) | 1.41 (1.06-1.92) |
| By BMI levels P for interaction=0.35 | |  |  |  |  |
| BMI<24 | Events/Sample (Incidence rate) | 111/2472 (5.60) | 29/476 (7.82) | 29/653 (6.20) | 125/1798 (8.91) |
|  | HR | 1.00 (Ref) | 1.33 (1.08-1.72) | 1.05 (0.77-1.42) | 1.59 (1.24-2.03) |
| BMI>=24 | Events/Sample (Incidence rate) | 58/892 (8.01) | 32/332 (10.95) | 21/261 (10.15) | 147/1660 (12.36) |
|  | HR | 1.00 (Ref) | 1.35 (1.07-1.88) | 1.17 (0.82-1.84) | 1.44 (1.13-1.96) |

**Supplementary Table 8**: The association between TyG, non-HDL cholesterol, remnant cholesterol and their joint effects with incident stroke risk **with complete case analysis** in CHARLS

|  |  | **Model 1** | **p-value** | **Model 2** | **p-value** |
| --- | --- | --- | --- | --- | --- |
| TyG | Below median  (Reference) | 1.00 |  | 1.00 |  |
|  | Above median | 1.58 (1.34-1.88) | <0.001 | 1.41 (1.18-1.69) | 0.01 |
| Non-HDL cholesterol | Below median  (Reference) | 1.00 |  | 1.00 |  |
|  | Above median | 1.57 (1.32-1.86) | <0.001 | 1.49 (1.25-1.77) | <0.001 |
| Remnant cholesterol | Below median  (Reference) | 1.00 |  | 1.00 |  |
|  | Above median | 1.37 (1.15-1.62) | <0.001 | 1.26 (1.06-1.50) | 0.01 |
| TyG and non-HDL cholesterol | Both below median  (Reference) | 1.00 |  | 1.00 |  |
|  | Only TyG above median | 1.44 (1.09-1.89) | 0.01 | 1.33 (1.01-1.76) | 0.04 |
|  | Only non-HDL cholesterol above median | 1.41 (1.08-1.85) | 0.01 | 1.44 (1.10-1.88) | 0.01 |
|  | Both above median | 1.98 (1.60-2.45) | <0.001 | 1.76 (1.41-2.19) | <0.001 |
| TyG and remnant cholesterol | Both below median  (Reference) | 1.00 |  | 1.00 |  |
|  | Only TyG above median | 1.40 (1.09-1.91) | 0.001 | 1.27 (1.01-1.57) | 0.03 |
|  | Only remnant cholesterol above median | 1.11 (0.81-1.52) | 0.52 | 1.07 (0.77-1.47) | 0.69 |
|  | Both above median | 1.63 (1.34-1.97) | <0.001 | 1.45 (1.18-1.77) | <0.001 |

**Supplementary Table 9**: The association between TyG, Non-HDL cholesterol, Remnant cholesterol and their joint effects with incident stroke risk **further adjusting for antihypertensive, antidiabetic and lipid-lowering drug use** in CHARLS

|  |  | **Model 1** | **p-value** | **Model 2** | **p-value** |
| --- | --- | --- | --- | --- | --- |
| TyG | Below median  (Reference) | 1.00 |  | 1.00 |  |
|  | Above median | 1.49 (1.25-1.77) | <0.001 | 1.42 (1.19-1.70) | <0.001 |
| Non-HDL cholesterol | Below median  (Reference) | 1.00 |  | 1.00 |  |
|  | Above median | 1.53 (1.28-1.82) | <0.001 | 1.49 (1.25-1.78) | <0.001 |
| Remnant cholesterol | Below median  (Reference) | 1.00 |  | 1.00 |  |
|  | Above median | 1.31 (1.10-1.56) | 0.002 | 1.27 (1.07-1.51) | 0.01 |
| TyG and non-HDL cholesterol | Both below median  (Reference) | 1.00 |  | 1.00 |  |
|  | Only TyG above median | 1.36 (1.03-1.76) | 0.03 | 1.34 (1.01-1.77) | 0.04 |
|  | Only non-HDL cholesterol above median | 1.41 (1.08-1.84) | 0.01 | 1.44 (1.11-1.89) | <0.001 |
|  | Both above median | 1.85 (1.49-2.20) | <0.001 | 1.76 (1.42-2.21) | <0.001 |
| TyG and remnant cholesterol | Both below median  (Reference) | 1.00 |  | 1.00 |  |
|  | Only TyG above median | 1.41 (1.07-1.94) | 0.01 | 1.41 (1.04-1.690) | 0.02 |
|  | Only remnant cholesterol above median | 1.10 (0.80-1.51) | 0.56 | 1.08 (0.79-1.47) | 0.63 |
|  | Both above median | 1.62 (1.31-1.96) | <0.001 | 1.56 (1.29-1.88) | <0.001 |

**Supplementary Table 10**: The association between TyG, Non-HDL cholesterol, Remnant cholesterol and their joint effects with incident stroke risk **excluding participants from the first two years of follow up** in CHARLS

|  |  | **Model 1** | **p-value** | **Model 2** | **p-value** |
| --- | --- | --- | --- | --- | --- |
| TyG | Below median  (Reference) | 1.00 |  | 1.00 |  |
|  | Above median | 1.48 (1.24-1.78) | <0.001 | 1.33 (1.08-1.69) | <0.001 |
| Non-HDL cholesterol | Below median  (Reference) | 1.00 |  | 1.00 |  |
|  | Above median | 1.55 (1.31-1.89) | <0.001 | 1.46 (1.21-1.78) | <0.001 |
| Remnant cholesterol | Below median  (Reference) | 1.00 |  | 1.00 |  |
|  | Above median | 1.38 (1.13-1.64) | <0.001 | 1.25 (1.04-1.51) | 0.02 |
| TyG and non-HDL cholesterol | Both below median  (Reference) | 1.00 |  | 1.00 |  |
|  | Only TyG above median | 1.39 (1.07-1.89) | <0.001 | 1.32 (1.07-1.78) | <0.001 |
|  | Only non-HDL cholesterol above median | 1.50 (1.12-1.96) | <0.001 | 1.49 (1.13-1.93) | <0.001 |
|  | Both above median | 1.91 (1.50-2.42) | <0.001 | 1.72 (1.29-2.09) | <0.001 |
| TyG and remnant cholesterol | Both below median  (Reference) | 1.00 |  | 1.00 |  |
|  | Only TyG above median | 1.38 (1.06-1.85) | 0.02 | 1.29 1.02-1.68) | 0.04 |
|  | Only remnant cholesterol above median | 1.12 (0.83-1.52) | 0.35 | 1.07 (0.78-1.51) | 0.62 |
|  | Both above median | 1.57 (1.27-1.93) | <0.001 | 1.46 (1.20-1.91) | <0.001 |

**Supplementary Table 11: The association between TyG, Non-HDL cholesterol, Remnant cholesterol and their joint effects with incident stroke risk with the competing risk model in CHARLS**

|  |  | **Model 1** | **p-value** | **Model 2** | **p-value** |
| --- | --- | --- | --- | --- | --- |
| TyG | Below median  (Reference) | 1.00 |  | 1.00 |  |
|  | Above median | 1.49 (1.24-1.79) | <0.001 | 1.38 (1.14-1.66) | <0.001 |
| Per 1-SD | | 1.22 (1.13-1.32) | <0.001 | 1.18 (1.08-1.28) | <0.001 |
| Non-HDL cholesterol | Below median  (Reference) | 1.00 |  | 1.00 |  |
|  | Above median | 1.47 (1.22-1.76) | <0.001 | 1.39 (1.15-1.67) | <0.001 |
| Per 1-SD | | 1.19 (1.10-1.28) | <0.001 | 1.15 (1.05-1.24) | <0.001 |
| Remnant cholesterol | Below median  (Reference) | 1.00 |  | 1.00 |  |
|  | Above median | 1.27 (1.07-1.52) | <0.001 | 1.21 (1.04-1.49) | 0.03 |
| Per 1-SD | | 1.12 (1.06-1.18) | <0.001 | 1.09 (1.02-1.16) | 0.01 |
| TyG and non-HDL cholesterol | Both below median  (Reference) | 1.00 |  | 1.00 |  |
|  | Only TyG above median | 1.39 (1.07-1.85) | 0.01 | 1.30 (0.97-1.74) | 0.17 |
|  | Only non-HDL cholesterol above median | 1.35 (1.04-1.86) | 0.01 | 1.29 (0.98-1.66) | 0.12 |
|  | Both above median | 1.87 (1.48-2.32) | <0.001 | 1.73 (1.37-2.18) | <0.001 |
| TyG and remnant cholesterol | Both below median  (Reference) | 1.00 |  | 1.00 |  |
|  | Only TyG above median | 1.50 (1.11-1.96) | <0.001 | 1.38 (1.04-1.87) | 0.03 |
|  | Only remnant cholesterol above median | 1.19 (0.91-1.60) | 0.35 | 1.16 (0.87-1.55) | 0.37 |
|  | Both above median | 1.58 (1.29-1.92) | <0.01 | 1.46 (1.17-1.81) | <0.001 |

**Supplementary Table 12**: The association between TyG, non-HDL cholesterol, remnant cholesterol and their joint effects with incident stroke risk **with tertiles used for TyG cut-offs** in CHARLS

|  |  | **Model 1** | **p-value** | **Model 2** | **p-value** |
| --- | --- | --- | --- | --- | --- |
| TyG | T1 (Reference group) | 1.00 |  | 1.00 |  |
|  | T2 | 1.68 (1.35-2.10) | <0.001 | 1.42 (1.14-1.89) | <0.001 |
|  | T3 | 1.83 (1.47-2.20) | <0.001 | 1.62 (1.29-2.04) | <0.001 |
| TyG and non-HDL cholesterol | TyG T1 Non-HDL C below median  (Reference) | 1.00 |  | 1.00 |  |
|  | TyG T2 Non-HDL C below median | 1.61 (1.19-2.18) | 0.002 | 1.54 (1.07-2.22) | 0.02 |
|  | TyG T3 Non-HDL C below median | 1.66 (1.16-2.38) | 0.003 | 1.59 (1.18-2.16) | <0.001 |
|  | TyG T1 Non-HDL C above median | 1.47 (1.03-2.12) | 0.02 | 1.51 (1.05-2.18) | 0.02 |
|  | TyG T2 Non-HDL C above median | 2.15 (1.65-2.81) | <0.001 | 1.97 (1.50-2.60) | <0.001 |
|  | TyG T3 Non-HDL C above median | 2.22 (1.71-2.90) | <0.001 | 2.11 (1.58-2.80) | <0.001 |
| TyG and remnant cholesterol | TyG T1 RC below median  (Reference) | 1.00 |  | 1.00 |  |
|  | TyG T2 RC below median | 1.53 (1.07-2.36) | 0.03 | 1.37 (0.93-2.04) | 0.39 |
|  | TyG T3 RC below median | 1.69 (1.30-2.21) | 0.001 | 1.60 (1.23-2.10) | <0.001 |
|  | TyG T1 RC above median | 1.36 (1.05-1.94) | 0.02 | 1.12 (0.83-1.56) | 0.61 |
|  | TyG T2 RC above median | 1.70 (1.29-2.24) | <0.001 | 1.65 (1.26-2.18) | <0.001 |
|  | TyG T3 Non-HDL C above median | 1.88 (1.48-2.37) | <0.001 | 1.74 (1.30-2.12) | <0.001 |

**Supplementary Table 13**: The association between TyG, Non-HDL cholesterol, Remnant cholesterol and their joint effects with incident stroke risk **with tertiles used for non-HDL cholesterol cut-offs** in CHARLS

|  |  | **Model 1** | **p-value** | **Model 2** | **p-value** |
| --- | --- | --- | --- | --- | --- |
| Non-HDL cholesterol | T1 (Reference group) | 1.00 |  | 1.00 |  |
|  | T2 | 1.38 (1.10-1.73) | <0.001 | 1.31 (1.04-1.44) | 0.01 |
|  | T3 | 1.71 (1.38-2.11) | <0.001 | 1.57 (1.27-1.95) | <0.001 |
| TyG and non-HDL cholesterol | Non-HDL cholesterol T1 TyG below median  (Reference) | 1.00 |  | 1.00 |  |
|  | Non-HDL cholesterol T2 TyG below median | 1.30 (0.96-1.76) | 0.09 | 1.27 (0.93-1.72) | 0.12 |
|  | Non-HDL cholesterol T3 TyG below median | 1.67 (1.19-2.35) | <0.001 | 1.65 (1.17-2.33) | <0.001 |
|  | Non-HDL cholesterol T1 TyG above median | 1.69 (1.17-2.23) | <0.001 | 1.56 (1.17-2.10) | <0.001 |
|  | Non-HDL cholesterol T2 TyG above median | 1.74 (1.23-2.35) | <0.001 | 1.61 (1.14-2.28) | <0.01 |
|  | Non-HDL cholesterol T3 TyG above median | 2.17 (1.68-2.80) | <0.001 | 1.92 (1.48-2.50) | <0.001 |

**Supplementary Table 14**: The association between TyG, Non-HDL cholesterol, Remnant cholesterol and their joint effects with incident stroke risk **with tertiles used for remnant cholesterol (RC) cut-offs** in CHARLS

|  |  | **Model 1** | **p-value** | **Model 2** | **p-value** |
| --- | --- | --- | --- | --- | --- |
| Remnant cholesterol | T1 (Reference group) | 1.00 |  | 1.00 |  |
|  | T2 | 1.36 (1.09-1.69) | 0.01 | 1.29 (1.04-1.62) | 0.02 |
|  | T3 | 1.77 (1.43-2.18) | <0.001 | 1.60 (1.29-1.98) | <0.001 |
| TyG and Remnant cholesterol | RC T1 TyG below median  (Reference) | 1.00 |  | 1.00 |  |
|  | RC T2 TyG below median | 1.26 (0.95-1.68) | 0.11 | 1.25 (0.94-1.66) | 0.13 |
|  | RC T3 TyG below median | 1.89(1.19-2.87) | <0.001 | 1.72 (1.09-2.51) | <0.001 |
|  | RC T1 TyG above median | 1.67 (1.13-2.05) | <0.001 | 1.51 (1.15-1.98) | 0.02 |
|  | RC T2 TyG above median | 1.72 (1.10-2.41) | <0.001 | 1.58 (1.10-1.94) | <0.001 |
|  | RC T3 TyG above median | 1.90 (1.51-2.38) | <0.001 | 1.71 (1.10-2.61) | <0.001 |

**Supplementary Table 15: Number and proportion of stroke events on exact date and survey-wave interview date in CHARLS 2011-2020.**

| **Exposure group** | **Category** | **Stroke events, n** | **Exact onset date available, n (%)** | **Survey-wave interview date used, n (%)** |
| --- | --- | --- | --- | --- |
| **TyG** | Below median | 219 | 139 (63.5) | 80 (36.5) |
|  | Above median | 333 | 211 (63.4) | 122 (36.6) |
| **Non-HDL cholesterol** | Below median | 215 | 135 (62.8) | 80 (37.2) |
|  | Above median | 337 | 208 (61.7) | 129 (38.3) |
| **Remnant cholesterol** | Below median | 230 | 140 (60.9) | 90 (39.1) |
|  | Above median | 322 | 207 (64.3) | 115 (35.7) |
| **TyG and non-HDL cholesterol** | Both below median | 129 | 79 (61.2) | 50 (38.8) |
|  | Only TyG above median | 86 | 52 (60.5) | 34 (39.5) |
|  | Only non-HDL cholesterol above median | 90 | 59 (65.6) | 31 (34.4) |
|  | Both above median | 247 | 161 (65.2) | 86 (34.8) |
| **TyG and remnant cholesterol** | Both below median | 169 | 107 (63.3) | 62 (36.7) |
|  | Only TyG above median | 61 | 40 (65.6) | 21 (34.4) |
|  | Only remnant cholesterol above median | 50 | 34 (68.0) | 16 (32.0) |
|  | Both above median | 272 | 166 (61.0) | 106 (39.0) |
